# MRI-Based Radiomics for Non-Invasive Prediction of Molecular Biomarkers in Gliomas

**DOI:** 10.3390/cancers18030491

**Published:** 2026-02-02

**Authors:** Edoardo Agosti, Karen Mapelli, Gianluca Grimod, Amedeo Piazza, Marco Maria Fontanella, Pier Paolo Panciani

**Affiliations:** 1 Division of Neurosurgery, Department of Medical and Surgical Specialties, Radiological Sciences and Public Health, University of Brescia, Piazzale Spedali Civili 1, 25123 Brescia, Italy; edoardo_agosti@libero.it (E.A.); k.mapelli@studenti.unibs.it (K.M.); marco.fontanella@unibs.it (M.M.F.); 2 Neurosurgery Unit, IRCCS San Matteo, 27100 Pavia, Italy; g.grimod@gmail.com; 3 Department of Neurosurgery, “Sapienza” University, 49911 Rome, Italy; amedeo.piazza@icloud.com

**Keywords:** radiomics, molecular biology, glioma, magnetic resonance imaging, machine learning, deep learning, systematic review

## Abstract

Background: Radiomics has emerged as a promising approach to non-invasively characterize the molecular landscape of gliomas, providing quantitative, high-dimensional data derived from routine MRI. Given the recent shift toward molecularly driven classification, radiomics may support precision oncology by predicting key genomic, epigenetic, and phenotypic alterations without the need for invasive tissue sampling. This systematic review aimed to synthesize current radiomics applications for the non-invasive prediction of molecular biomarkers in gliomas, evaluating methodological trends, performance metrics, and translational readiness. Methods: This review followed the PRISMA 2020 guidelines. A systematic search was conducted in PubMed, Ovid MEDLINE, and Scopus on 10 January 2025, and updated on 1 February 2025, using predefined MeSH terms and keywords related to glioma, radiomics, machine learning, deep learning, and molecular biomarkers. Eligible studies included original research using MRI-based radiomics to predict molecular alterations in human gliomas, with reported performance metrics. Data extraction covered study design, cohort size, MRI sequences, segmentation approaches, feature extraction software, computational methods, biomarkers assessed, and diagnostic performance. Methodological quality was evaluated using the Radiomics Quality Score (RQS), Image Biomarker Standardization Initiative (IBSI) criteria, and Newcastle–Ottawa Scale (NOS). Due to heterogeneity, no meta-analysis was performed. Results: Of 744 screened records, 70 studies met the inclusion criteria. A total of 10,324 patients were included across all studies (mean 140 patients/study, range 23–628). The most frequently employed MRI sequences were T2-weighted (59 studies, 84.3%), contrast-enhanced T1WI (53 studies, 75.7%), T1WI (50 studies, 71.4%), and FLAIR (48 studies, 68.6%); diffusion-weighted imaging was used in only 7 studies (12.8%). Manual segmentation predominated (52 studies, 74.3%), whereas automated approaches were used in 13 studies (18.6%). Common feature extraction platforms included 3D Slicer (20 studies, 28.6%) and MATLAB-based tools (17 studies, 24.3%). Machine learning methods were applied in 47 studies (67.1%), with support vector machines used in 29 studies (41.4%); deep learning models were implemented in 27 studies (38.6%), primarily convolutional neural networks (20 studies, 28.6%). IDH mutation was the most frequently predicted biomarker (49 studies, 70%), followed by ATRX (27 studies, 38.6%), MGMT methylation (8 studies, 11,4%), and 1p/19q codeletion (7 studies, 10%). Reported AUC values ranged from 0.80 to 0.99 for IDH, approximately 0.71–0.953 for 1p/19q, 0.72–0.93 for MGMT, and 0.76–0.97 for ATRX, with deep learning or hybrid pipelines generally achieving the highest performance. RQS values highlighted substantial methodological variability, and IBSI adherence was inconsistent. NOS scores indicated high-quality methodology in a limited subset of studies. Conclusions: Radiomics demonstrates strong potential for the non-invasive prediction of key glioma molecular biomarkers, achieving high diagnostic performance across diverse computational approaches. However, widespread clinical translation remains hindered by heterogeneous imaging protocols, limited standardization, insufficient external validation, and variable methodological rigor.

## Simple Summary

Radiomics for molecular characterization of gliomas demonstrated high diagnostic accuracy, particularly for IDH mutations (AUC 0.80–0.99) and ATRX (AUC 0.76–0.97). While machine learning and deep learning models showed superior results, the field is characterized by a high reliance on manual segmentation and variable methodological standardization. Although radiomics is a robust tool for non-invasive biomarker prediction, clinical integration is currently hindered by imaging heterogeneity and the need for standardized, prospective external validation.

## 1. Introduction

Gliomas represent the most common and aggressive primary brain tumors (PBTs), originating from glial cells within the central nervous system (CNS). They account for approximately 25–30% of all CNS malignancies and nearly 80% of malignant cases. Their biological behavior spans a wide spectrum, from indolent low-grade astrocytomas to glioblastomas characterized by rapid progression, therapeutic resistance, and a dismal prognosis. Despite advances in neurosurgery, radiotherapy, and targeted therapies, overall survival for high-grade cases remains poor. The infiltrative growth, marked intratumoral heterogeneity, and inevitable recurrence severely limit current treatment effectiveness [1,2,3,4,5,6].

Imaging is pivotal throughout the diagnostic and therapeutic pathway. While conventional MRI remains the mainstay for detection and surgical planning, advanced techniques—such as DWI, PWI, and MRS—have expanded the assessment of tumor cellularity and metabolic profiles. However, MRI still faces challenges in capturing full biological complexity. Intratumoral heterogeneity often surpasses human interpretative limits, while interobserver variability contributes to diagnostic uncertainty. Consequently, standard imaging provides only an indirect representation of the underlying tumor biology [7,8,9].

The diagnostic paradigm has recently shifted from purely histopathological classification toward a molecularly driven framework, now fundamental in the WHO classification. Key alterations—including IDH status, 1p/19q codeletion, MGMT methylation, ATRX loss, TERT mutations, EGFR amplification, and histone H3 mutations—underpin current grading and therapeutic decisions [10,11,12]. Although these markers refine prognostic assessment, their detection currently necessitates invasive biopsy or resection. Such procedures are not always feasible due to tumor location or surgical risk and may suffer from sampling bias. This underscores the need for reliable, non-invasive approaches to monitor the molecular landscape [13,14].

**Figure 1:**
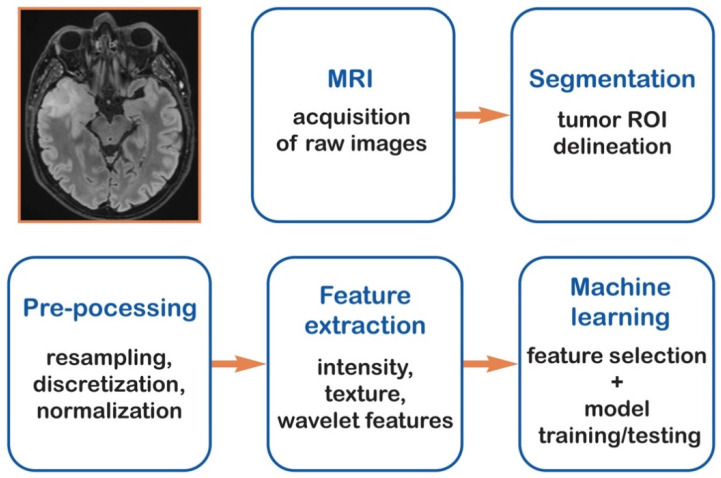
Graphical representation of radiomics workflow.

In this context, radiomics has emerged as a quantitative tool to bridge the gap between imaging and biology. This high-throughput extraction of features transforms qualitative visuals into quantifiable data, describing shape, intensity, and texture patterns imperceptible to the eye [15,16]. A typical workflow encompasses acquisition, preprocessing, segmentation, and model construction (Figure 1).

When integrated with artificial intelligence (AI), particularly machine learning (ML) and deep learning (DL), these models support clinical decision-making. Over the past decade, the field has grown significantly, driven by computational power and standardized pipelines. Methodological innovations, such as deep learning-based recognition and habitat imaging, have further refined the characterization of distinct intratumoral subregions. One of the most compelling applications is the non-invasive prediction of molecular alterations traditionally assessed via tissue sampling [17]. Preoperative MRI models have demonstrated substantial accuracy in predicting IDH and 1p/19q status, alongside markers like p53, PTEN, TERT, ATRX, VEGF, EGFR, Ki-67, and MGMT, with AUC values ranging from 0.70 to 0.92 [18,19,20].

The clinical implications are profound: non-invasively inferring molecular profiles could enhance risk stratification and treatment selection. Beyond classification, these tools show promise in forecasting therapeutic sensitivity and survival outcomes. By enabling longitudinal monitoring, they provide insights into tumor evolution and early recurrence indicators [21,22]. Importantly, this approach is reproducible and leverages routinely acquired clinical images without additional interventions.

Despite this evidence, translation into routine practice remains limited by a lack of standardized protocols, feature extraction variability, and the need for transparent AI. Moreover, the literature remains fragmented, lacking a comprehensive synthesis of the synergistic relationship between imaging and molecular biology [23,24]. While general reviews exist, dedicated evaluations of this integration in gliomas are currently limited to a few studies [25,26,27,28,29]. This convergence represents a paradigmatic shift, transforming imaging into a potential surrogate biomarker. Tailoring decisions to individual biological characteristics aligns with the principles of precision oncology [30]. This systematic review aims to evaluate the current state of radiomics-based profiling, critically assess methodological quality, and highlight future directions.

## 2. Materials and Methods

### 2.1. Literature Review

This systematic review was conducted in accordance with the Preferred Reporting Items for Systematic Reviews and Meta-Analyses (PRISMA) guidelines [31]. Two independent investigators (E.A. and K.M.) performed a comprehensive and structured search of the scientific literature using the PubMed, Ovid MEDLINE, and Scopus databases. The initial search was conducted on 10 January 2025, with a final update performed on 1 February 2025.

A comprehensive search strategy was developed using a combination of controlled vocabulary (MeSH terms) and free-text keywords related to radiomics, molecular biology, and glioma. The primary search terms included: “glioma,” “glioblastoma,” “radiomics,” “radiogenomics,” “machine learning,” “deep learning,” “molecular biomarkers,” “IDH,” “1p/19q,” “MGMT,” “TERT,” “ATRX,” and “EGFR.” Boolean operators were applied as follows: (glioma OR glioblastoma) AND (radiomics OR radiogenomics) AND (molecular biology OR molecular biomarkers OR IDH OR 1p/19q OR MGMT OR TERT OR ATRX OR EGFR). Additional eligible studies were identified through a manual screening of the reference lists of selected articles and relevant review papers.

The inclusion criteria were defined as follows: only studies published in English were considered eligible. We included original research articles specifically investigating the application of radiomics for the non-invasive prediction of molecular biomarkers in gliomas. Eligible studies were required to be based on MRI-derived radiomic features and to employ machine learning (ML) and/or deep learning (DL) models. Furthermore, only studies reporting quantitative performance metrics, such as accuracy, area under the curve (AUC), sensitivity, or specificity, were included. The exclusion criteria comprised editorials, letters, narrative reviews, systematic reviews, meta-analyses, case reports, and conference abstracts. Studies not specifically focused on gliomas were excluded, as well as those not involving radiomic feature extraction from MRI. Additionally, studies lacking molecular ground truth validation or those without a clearly defined methodology and/or performance outcomes were excluded from the analysis.

All retrieved references were imported into EndNote X9 (Clarivate Analytics, Philadelphia, PA, USA), where duplicate records were automatically and manually removed. The screening process was conducted independently by the two reviewers based on titles and abstracts according to the predefined eligibility criteria. Discrepancies were resolved by consensus or, when necessary, by consultation with a third senior reviewer (P.P.P.). Articles deemed eligible underwent full-text assessment for final inclusion.

### 2.2. Data Extraction

Data extraction was independently performed by two reviewers (E.A. and K.M.) using a standardized and predefined extraction template to ensure methodological consistency and reproducibility. For each eligible study, detailed information was systematically collected regarding the authorship and year of publication, as well as the total cohort size, including the distribution of cases into training, validation, and testing datasets.

Technical imaging parameters were recorded for each study, including the MRI sequences employed, specifically T1-weighted imaging, contrast-enhanced T1-weighted imaging (T1-CE), T2-weighted imaging, fluid-attenuated inversion recovery (FLAIR), dynamic susceptibility contrast perfusion imaging (DSC), and diffusion-weighted imaging (DWI). The segmentation approach adopted in each study was documented and categorized as manual, semi-automatic, or fully automatic. Information regarding the software platforms used for radiomic feature extraction, such as MATLAB, ImageJ, Pyradiomics, MaZda, IBEX, and 3D Slicer, was also collected. All available software versions were considered. Furthermore, the type of machine learning and/or deep learning models applied, including but not limited to support vector machines, random forests, logistic regression, convolutional neural networks, and ensemble models, was systematically recorded.

About molecular biology, the specific molecular patterns investigated were extracted, including IDH mutation status, 1p/19q codeletion, MGMT promoter methylation, ATRX mutation, TERT promoter mutation, EGFR amplification, p53 mutation, and Ki-67 expression. Finally, the diagnostic and predictive performance of each model was collected in terms of accuracy, area under the receiver operating characteristic curve (AUC), sensitivity, specificity, and other available performance metrics. AUC values indicate the model’s accuracy: 0.5 means no better than chance, 0.7 to 0.8 is considered acceptable, and values above 0.8 are generally required for potential clinical use.

When essential methodological or outcome data were unclear or missing, supplementary materials were consulted whenever available. Any disagreement between the two reviewers during the data extraction process was resolved by discussion and, if necessary, by consultation with the third senior reviewer (P.P.P.).

### 2.3. Outcomes

The primary outcome of this systematic review was to comprehensively characterize the current applications of radiomics for the non-invasive prediction of molecular biomarkers in gliomas, with particular emphasis on the most clinically relevant genomic and epigenetic alterations.

The secondary outcomes included the evaluation of the diagnostic and predictive performance of machine learning and deep learning models across different molecular targets, the assessment of the impact of MRI sequences and radiomic feature extraction pipelines on model accuracy, and the comparison between manual and automatic segmentation strategies. Additional secondary outcomes involved the identification of methodological trends, strengths, and limitations within the existing literature, as well as the assessment of the translational readiness of radiomics for integration into routine neuro-oncological practice.

### 2.4. Radiomics Quality Assessment

To ensure a transparent evaluation of the evidence, we assessed the methodological and technical quality of the included studies using two specific tools. The Radiomics Quality Score (RQS) was employed to measure the clinical and technical rigor of the radiomic workflows, while the Image Biomarker Standardization Initiative (IBSI) guidelines were used to evaluate the reproducibility and standardization of feature extraction.

A qualitative methodological assessment was performed using the RQS [32] framework with the aim of critically analyzing and comparing the clinical applicability, methodological robustness, and translational potential of the radiomic models developed in the included studies. The RQS was independently applied by two reviewers to evaluate key methodological domains, including imaging protocol quality, feature robustness, biological and clinical validation, model performance assessment, and data transparency.

In addition to the RQS evaluation, study reproducibility and technical rigor were further assessed according to the IBSI compliance checklist [33]. This evaluation was specifically focused on image pre-processing and radiomic feature extraction steps to verify adherence to standardized radiomics workflows. As several items of the IBSI checklist overlap with those included in the RQS framework, only the IBSI items specifically related to image pre-processing and technical reproducibility were considered in the final assessment.

The maximum attainable RQS is 36, with higher scores reflecting superior methodological quality and greater translational readiness. Studies were categorized as low quality when the RQS was below 30%, moderate quality when it ranged between 30% and 60%, and high quality when it exceeded 60%. Any discrepancies between reviewers in both RQS and IBSI evaluations were resolved through discussion and, when necessary, by consultation with a third senior reviewer.

### 2.5. Risk of Bias Assessment

**Figure 2:**
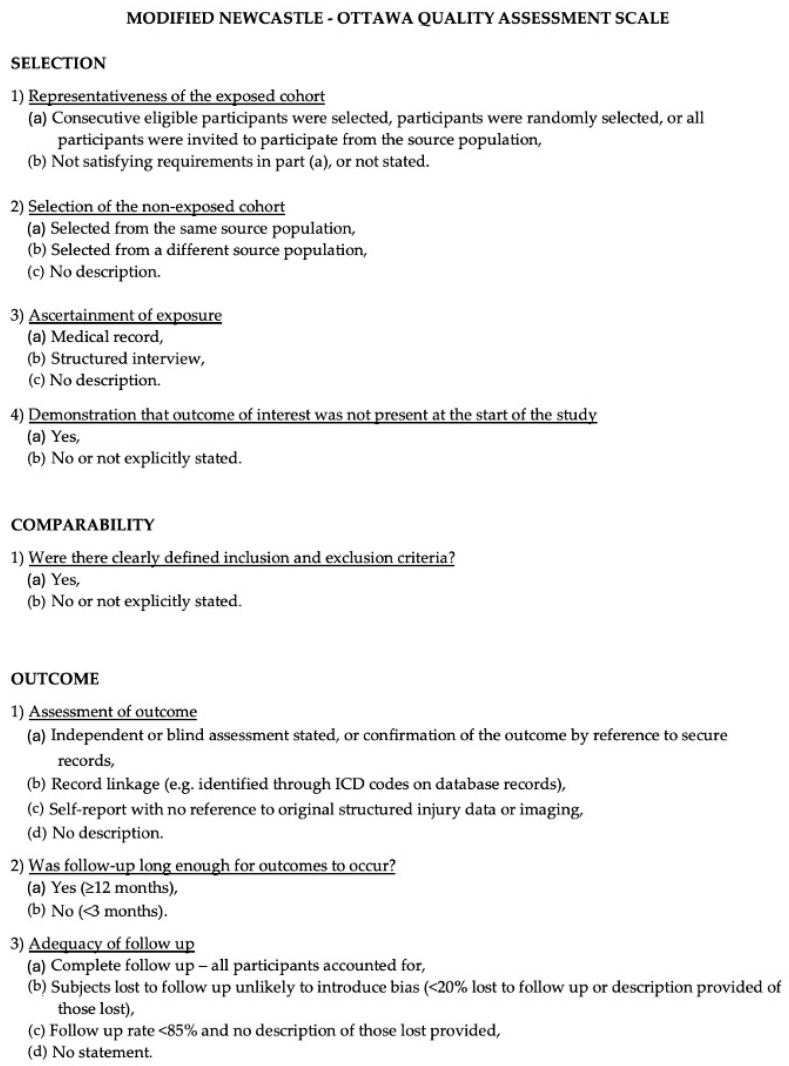
The Modified NOS.

The methodological quality and risk of bias of the included studies were assessed using the Newcastle–Ottawa Scale (NOS) [34], which evaluates non-randomized studies based on three main domains: selection of the study groups, comparability of the cohorts, and assessment of outcomes. Quality appraisal was conducted according to these predefined criteria, with a maximum achievable score of 9 points. Higher scores indicated superior methodological quality, and studies achieving a score of 7 or higher were classified as high quality. The risk of bias assessment was independently performed by two authors (E.A. and K.M.), and any discrepancies were resolved through re-evaluation and discussion with the involvement of a third senior reviewer (P.P.P.). The overall quality assessment is summarized in Figure 2.

### 2.6. Statistical Analysis

Descriptive statistics were used to summarize the characteristics of the included studies, including cohort size, MRI sequences utilized, segmentation methods, software platforms, molecular targets, and machine learning or deep learning models. Continuous variables were reported as ranges and medians, while categorical variables were expressed as absolute frequencies and percentages. Due to the substantial heterogeneity in imaging acquisition protocols, radiomic pipelines, molecular targets, and outcome reporting, a formal quantitative meta-analysis was not performed. Instead, a structured qualitative synthesis of the findings was conducted.

## 3. Results

### 3.1. PRISMA

**Figure 3:**
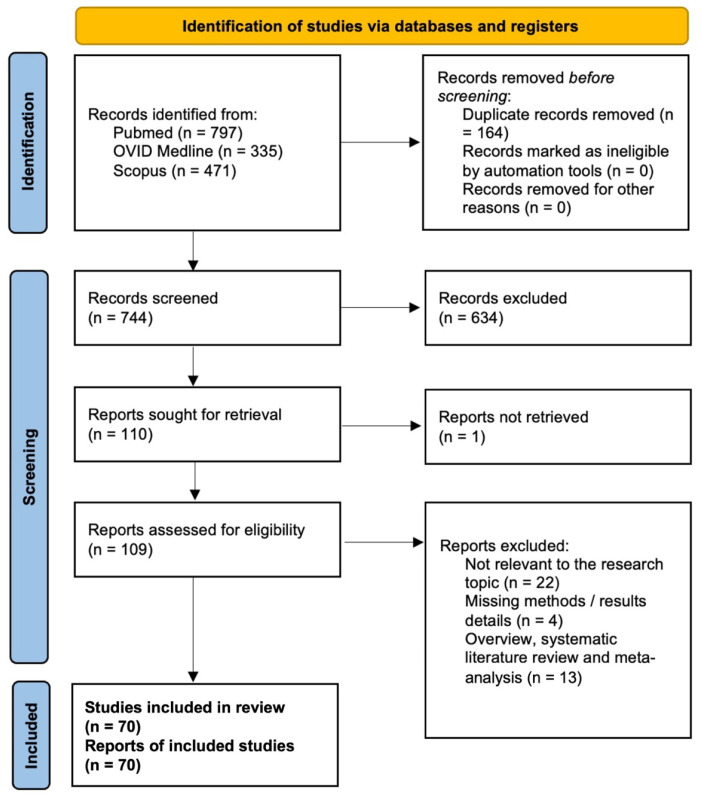
PRISMA flow chart (PROSPERO ID: CRD420251267827).

After removing duplicate records, a total of 744 studies were identified. Screening of titles and abstracts reduced this number to 109 articles eligible for full-text assessment. Of these, 70 studies met all inclusion criteria. The remaining 39 articles were excluded for the following reasons: 22 were not relevant to the research topic, 13 were systematic reviews or meta-analyses, and 4 lacked sufficient methodological details or results. The PRISMA flow diagram summarizing the selection process is presented in Figure 3.

**Figure A1:**
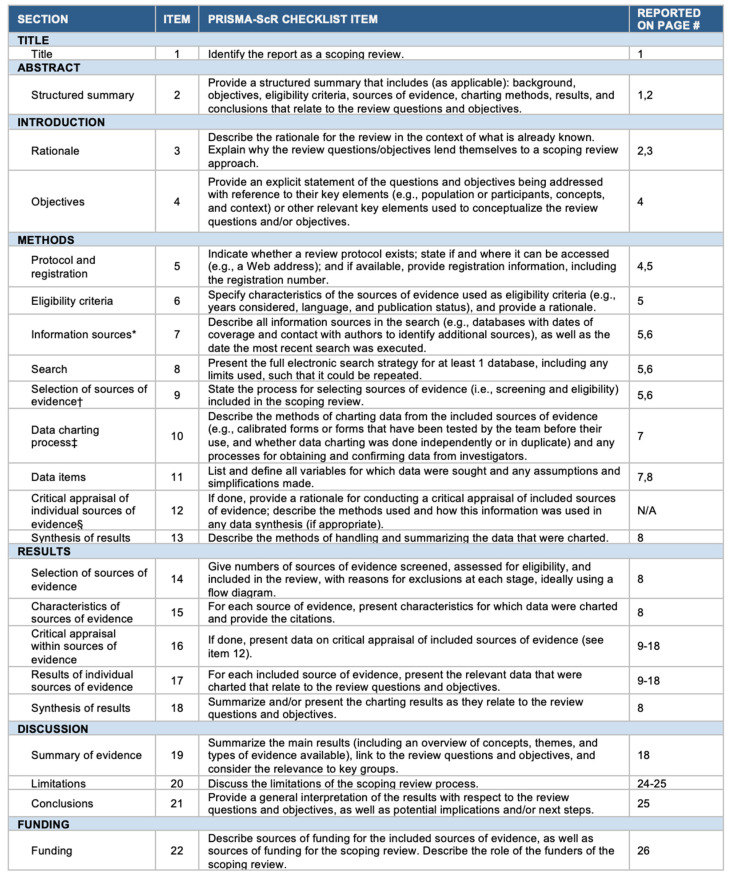
The PRISMA-ScR checklist (PROSPERO ID: CRD420251267827).PRISMA-ScR = Preferred Reporting Items for Systematic reviews and Meta-Analyses extension for Scoping Reviews. * Where sources of evidence (see second footnote) are compiled from, such as bibliographic databases, social media platforms, and Web sites. † A more inclusive/heterogeneous term used to account for the different types of evidence or data sources (e.g., quantitative and/or qualitative research, expert opinion, and policy documents) that may be eligible in a scoping review as opposed to only studies. This is not to be confused with information sources (see first footnote). ‡ The frameworks by Arksey and O’Malley (6) and Levac and colleagues (7) and the Joanna Briggs Institute guidance (4, 5) refer to the process of data extraction in a scoping review as data charting. § The process of systematically examining research evidence to assess its validity, results, and relevance before using it to inform a decision. This term is used for items 12 and 19 instead of “risk of bias” (which is more applicable to systematic reviews of interventions) to include and acknowledge the various sources of evidence that may be used in a scoping review (e.g., quantitative and/or qualitative research, expert opinion, and policy document).

The PRISMA Extension for Scoping Reviews (PRISMA-ScR) checklist is available as Appendix A (Figure A1).

### 3.2. Data Analysis

A summary of the included studies reporting on radiomics applications for the non-invasive prediction of molecular biomarkers in gliomas is presented in Table 1.

A total of 70 radiomics studies exploring molecular biomarkers in gliomas were published between 2017 and 2025. Publication volume increased progressively over time, with the highest concentration of studies appearing in 2020 (*n* = 13, 18.6%), followed by 2019 (*n* = 11, 15.7%), and 2021 (*n* = 10, 14.3%). The combined cohort across all studies included 10,324 patients, corresponding to a mean sample size of 140 patients per study (range 23–628). When available, aggregated training, validation, and testing cohorts included 7052 patients (68.3%), 2148 patients (20.8%), and 1124 patients (10.9%), respectively.

Across the included studies, MRI acquisition protocols showed substantial heterogeneity. The most frequently used sequence was T2-weighted imaging, reported in 59 of 70 studies (84.3%), followed closely by T1-contrast-enhanced (T1-CE), used in 53 studies (75.7%), and T1-weighted non-enhanced sequences, present in 50 studies (71.4%). FLAIR imaging was similarly common, appearing in 48 studies (68.6%), while diffusion-weighted imaging (DWI) was employed in a significantly smaller subset (*n* = 7, 10.0%). Advanced diffusion-derived maps, including DWI or connectomics, appeared in 4 studies (5.7%).

Segmentation methods were predominantly manual, used in 52 studies (74.3%), whereas semi-automated segmentation was reported in 9 studies (12.9%) and automated pipelines in 13 studies (18.6%). Regarding software, 3D Slicer represented the most frequently used platform (*n* = 20, 28.6%), followed by MATLAB-based environments (*n* = 17, 24.3%), BraTS/ITK-SNAP pipelines (*n* = 13, 18.6%), and custom deep-learning frameworks or unspecified in-house software (*n* = 24, 34.3%).

Machine learning techniques were applied in 47 studies (67.1%), with Support Vector Machines (SVM) representing the most common classifier (*n* = 29, 41.4%). Logistic regression models were used in 11 studies (15.7%), LASSO feature selection in 13 studies (18.6%), and Elastic Net regularization in 9 studies (12.9%). Deep learning architectures were implemented in 27 studies (38.6%), predominantly via Convolutional Neural Networks (CNNs) (*n* = 20, 28.6%), including ResNet-, DenseNet-, and U-Net–derived architectures. Transformer-based models appeared in 4 studies (5.7%), while radiomics–DL fusion approaches were reported in 6 studies (8.6%).

The most extensively investigated molecular biomarker was IDH mutation status, assessed in 49 studies (70.0%). ATRX was the second most frequently explored marker (*n* = 27, 38.6%), followed by MGMT promoter methylation (*n* = 8, 11.4%), 1p/19q codeletion (*n* = 7, 10.0%), and EGFR alterations (*n* = 4, 5.7%). Less commonly investigated markers included Ki-67 (*n* = 3, 4.3%), H3K27M (*n* = 3, 4.3%), VEGF (*n* = 2, 2.9%), TERT (*n* = 1, 1.4%), and PTEN (*n* = 1, 1.4%). Many studies investigated multiple markers simultaneously, particularly IDH in combination with 1p/19q and ATRX.

Performance assessment relied primarily on AUC, reported in 61 studies (87.1%), while accuracy was reported in 36 studies (51.4%), sensitivity and specificity in 20 studies (28.6%), and F1-scores in 8 studies (11.4%). Across all biomarkers and modeling strategies, the mean AUC for training datasets was 0.892, with mean AUCs of 0.864 for validation and 0.842 for testing cohorts, indicating consistent generalizability across studies.

Notably, IDH prediction achieved AUC values ranging from 0.80 to 0.99, with 3D Dense-UNet architectures providing the highest reported performance. Prediction of 1p/19q codeletion showed mean AUC values around 0.88 (range 0.71–0.953). MGMT methylation models reached AUCs between 0.72 and 0.93, particularly when hybrid ML–DL frameworks were applied. ATRX prediction exhibited higher variability, with AUCs spanning 0.76–0.97, reflecting differences in segmentation strategies and feature engineering. Emerging biomarkers showed promising performance, such as H3K27M, achieving AUCs up to 0.91, especially when incorporating diffusion-derived or connectomic features.

While the volume of primary research has expanded, a focused and systematic evaluation specifically dedicated to the integration of radiomics and molecular biology in gliomas remains limited to a small number of studies. To address this gap, we have synthesized the current landscape of the field in Table 2, contrasting the abundance of primary data with the scarcity of structured systematic assessments.

### 3.3. Handcrafted Radiomics and Deep Learning

Analysis of extraction methodologies revealed a divergence between handcrafted radiomics and deep learning (DL). Handcrafted approaches—utilizing expert-engineered features (e.g., shape, texture)—predominated in 63.5% of studies. Conversely, 36.5% of the literature employed DL architectures to extract high-dimensional latent representations directly from raw data. Table 3 provides a comparative breakdown of these paradigms, highlighting that while handcrafted features offer greater interpretability, DL-based models frequently captured complex patterns associated with higher predictive performance.

### 3.4. RQS and IBSI Assessment

The RQS and the IBSI compliance checklist were systematically applied to all 70 included studies. The detailed numerical RQS values and IBSI compliance results for each individual study are reported in Table 4.

### 3.5. NOS Assessment

The NOS was systematically applied to all 70 included studies to assess their methodological quality and risk of bias. The detailed numerical NOS scores for each individual study are reported in Table 5.

### 3.6. Descriptive Summary of Methodological and Performance Metrics

Table 6 summarizes the core metrics of the analyzed studies, highlighting NOS scores between 7 and 9 and the prevalence of manual segmentation at 70.3%. It further details the distribution of Deep Learning usage (27.0%) compared to handcrafted radiomics, alongside AUC performance values and biomarker focus.

## 4. Discussion

Radiomics and deep learning (DL) have emerged as transformative technologies for the non-invasive profiling of gliomas, offering a viable adjunct to biopsy-based tissue characterization. Across the 70 studies synthesized in this review, these frameworks demonstrated robust diagnostic accuracy in predicting cornerstone biomarkers, including IDH mutation, 1p/19q codeletion, and MGMT methylation. Despite substantial methodological variation, quantitative imaging effectively decodes spatial phenotypes that reflect the complex underlying tumor biology. Although some studies in the literature have attempted a meta-analysis, the high heterogeneity in imaging protocols, software tools, and clinical outcomes in our selection made a qualitative synthesis more appropriate to avoid misleading results.

In our review, T1-weighted, T2-weighted, and FLAIR sequences were the most frequently utilized for radiomic analysis. These sequences are widely preferred because they are highly standardized and less prone to the artifacts that affect more advanced techniques. In contrast, Diffusion-Weighted Imaging (DWI) was rarely used, likely due to technical challenges such as susceptibility artifacts and the lack of standardized b-values. While DWI is clinically essential, these hurdles often lead researchers to rely on more stable morphological sequences for radiomic pipelines.

### 4.1. Radiomics Application for Non-Invasive Molecular Profiling

#### 4.1.1. IDH Mutation

The detection of IDH mutation represents the most extensively investigated application in this field, with a breadth of evidence that allows for nuanced interpretations of performance and generalizability. Foundational works [35,36,39,40] established the conceptual groundwork by demonstrating that handcrafted texture features derived from conventional MRI could discriminate mutant from wildtype tumors. Although initially constrained by small sample sizes, these models proved that microstructural alterations induced by IDH mutations yield measurable signatures on routine imaging.

Refinement of these handcrafted pipelines showed AUCs ranging from 0.80 to 0.96 across various machine-learning classifiers [38,48], reinforcing the stability of IDH-related radiomic phenotypes across independent datasets. A pivotal milestone was achieved by Chang et al. [41], who utilized multi-sequence MRI and rigorous cross-cohort validation to reach AUCs of 0.90–0.94.

The robustness of these findings was corroborated by Nalawade et al. [58], who demonstrated high performance (AUCs 0.95 and 0.86) across independent datasets, illustrating the capacity of radiomics to generalize despite inter-institutional variations in acquisition. Methodological diversity was further enriched by studies experimenting with alternative extraction strategies, dimensionality-reduction techniques, and varied classifier architectures [52,53,55,56].

By 2020, evidence confirmed that radiomic signatures retain predictive value across diverse sequences, segmentation strategies, and population characteristics [64,66,67,70,71,74,75]. A notable advancement occurred with the transition to deep learning; Yogananda et al. [76] reported a landmark AUC of 0.99, one of the highest recorded for IDH prediction. This performance underscores that the biological influence of IDH mutation on cellularity and intratumoral heterogeneity produces distinguishable phenotypes uniquely suited to non-linear modeling.

Recent investigations have continued to affirm the efficacy of imaging-based classification. Studies by Huang et al. [79], Peng et al. [82], Santinha et al. [83], Verduin et al. [85], and Zhang et al. [102] refined earlier workflows by incorporating automated segmentation and advanced feature selection. These efforts indicate that the shift toward hybrid or deep radiomics yields steady improvements in model robustness. A substantial contribution from Liang et al. [97] demonstrated the potential of integrative frameworks by simultaneously predicting IDH status and the Ki-67 proliferation index (AUCs 0.97 and 0.93). This dual-prediction approach highlights the ability of radiomics to infer multilayered biological information from imaging.

The most recent progress in this domain was presented by Su et al. [104], who introduced a next-generation hybrid framework integrating radiomics, deep learning, and EGFR-related signatures. Their reported AUCs of 0.943 and 0.912 across separate cohorts illustrate not only high accuracy but also improved stability across different patient populations.

#### 4.1.2. 1p/19q Codeletion

Although evaluated in fewer studies than IDH mutation, the prediction of 1p/19q codeletion has yielded encouraging diagnostic results while identifying key methodological challenges. Initial radiomics models focused on handcrafted texture and intensity features from conventional MRI. For instance, Kim et al. [54] reported an AUC of 0.71, a modest outcome that underscores the difficulty of identifying chromosomal arm alterations through standard imaging alone.

A more sophisticated strategy was implemented by Chang et al. [42], who developed a dual-prediction model for IDH and 1p/19q status. This approach achieved an AUC of 0.88, a significant advancement over earlier findings. The improved performance likely stems from the biological enrichment of codeletion within IDH-mutant tumors, allowing the model to leverage shared imaging signatures. Furthermore, the use of multi-sequence MRI and a broader feature set enhanced the detection of diffuse infiltration and architectural patterns characteristic of the oligodendroglial lineage.

Consistent results were documented by Decuyper et al. [66], who achieved an AUC of 0.87 using machine-learning classifiers on quantitative MRI data. Their work confirms the reproducibility of 1p/19q-related signatures across independent cohorts. Crucially, they emphasized that robust normalization and feature harmonization are vital to mitigate the impact of inter-scanner variability on predictive stability.

Deep learning (DL) has further elevated performance benchmarks for this biomarker. Yogananda et al. [76] achieved an AUC of 0.953 by employing a convolutional neural network (CNN) on multi-parametric MRI. Unlike traditional pipelines, this DL model extracts hierarchical representations directly from the data, capturing intensity gradients and microstructural heterogeneity that often elude handcrafted descriptors. This jump in accuracy suggests that the 1p/19q phenotype consists of complex spatial patterns best characterized by non-linear modeling.

Recent contributions continue to expand the empirical foundation of this field. Fan et al. [77] reached an AUC of 0.8079 using multi-sequence radiomic signatures. While slightly lower than the metrics reported by Chang et al. [42] or Decuyper et al. [66], their results confirm that non-invasive prediction remains feasible across varied protocols and populations. Discrepancies in reported AUCs may be attributed to differences in cohort size, field strength, and segmentation techniques, reinforcing the need for standardized pipelines in multicenter research.

Finally, the work of Medeiros et al. [91] reflects the transition toward comprehensive profiling. By incorporating multi-parametric sequences—including FLAIR, contrast-enhanced T1, and diffusion metrics—this approach seeks to more accurately mirror the tumor’s underlying genomic architecture through holistic radiomic analysis.

#### 4.1.3. p53

Radiomic prediction of p53 mutation remains an emerging area of study. Li et al. [46] provided one of the primary systematic analyses, achieving a training AUC of 0.896 and a validation AUC of 0.763. While few studies target p53 exclusively, broader radiogenomic investigations—including those by Liang et al. [48], Han et al. [53], and Peng et al. [82]—indirectly support the feasibility of identifying features linked to genomic instability. These findings suggest that p53-related phenotypes may manifest as specific texture heterogeneities and structural irregularities. Existing evidence indicates that integrated multi-marker or deep learning frameworks are likely required to enhance the detectability of this status.

#### 4.1.4. PTEN

The characterization of PTEN loss is currently less extensively documented. Following an initial exploration by Li et al. [57], broader investigations [39,75,85] have observed that radiomic features related to tumor infiltration, edema extension, and microstructural disruption can reflect the PI3K/AKT dysregulation pathway. These correlations provide biological plausibility for the non-invasive prediction of PTEN alterations.

#### 4.1.5. TERT Promoter Mutation

Fang et al. [78] reported encouraging performance for TERT promoter mutation detection, with an AUC of 0.8446 and 80% accuracy. Subsequent research into markers with shared biological pathways [53,61,92] has highlighted that proliferative and genomic-stability alterations produce quantifiable imaging patterns. Furthermore, Zhang et al. [102] utilized a multi-target framework to incorporate perfusion and heterogeneity features specifically aligned with TERT-driven tumor behavior.

#### 4.1.6. ATRX

ATRX has evolved into a cornerstone of radiogenomic research. Early texture-based models and classical machine-learning classifiers utilized by Chaddad et al. [51], Haubold et al. [68], Calabrese et al. [63,86], and Kihira et al. [80] consistently demonstrated high predictive accuracy. While Li et al. [47] noted substantial internal AUCs (0.94), they also identified cross-center variability as a primary challenge when validation scores dropped to 0.725.

Recent studies have addressed these limitations by increasing cohort sizes and methodological rigor [59,84,87,88,90,92,94,96]. Ma et al. [90] achieved stable AUCs (0.76–0.84) across multiple datasets, while Lin et al. [98] reported landmark AUCs of 0.97 (training) and 0.91 (testing). The subsequent integration of deep learning and multi-sequence strategies [97,99] has confirmed ATRX as one of the most reproducible biomarkers in the current literature.

#### 4.1.7. VEGF

Initial assessment of VEGF prediction by Sun et al. [60] yielded a meaningful validation AUC of 0.702. Further support for these correlates is found in studies examining angiogenesis-linked markers or perfusion-driven radiomics [67,81,83]. Zhang et al. [102] subsequently integrated these features into a comprehensive multi-marker model to refine predictive performance.

#### 4.1.8. EGFR

Predictive frameworks for EGFR status have shown that tumor proliferation and metabolic remodeling generate detectable phenotypes. Li et al. [45] achieved strong results (validation AUC 0.95), while additional radiogenomic studies [64,72,74] reinforce the ability of radiomics to capture structural patterns related to the EGFR pathway. Su et al. [104] recently demonstrated that dual-marker architectures (IDH/EGFR) can effectively model proliferative dynamics across independent cohorts.

#### 4.1.9. Ki-67

Li et al. [37] first explored Ki-67 prediction, achieving 83% accuracy and an AUC of 0.781. Indirect support for capturing growth kinetics is provided by studies on overlapping proliferative markers [39,40,79]. Notably, Liang et al. [97] significantly advanced the field with a dual-marker model that predicted both IDH and Ki-67 with high accuracy (AUCs 0.97 and 0.93), validating the robustness of these signatures across various modeling strategies.

#### 4.1.10. MGMT Methylation

The reliability of MGMT signatures varies across the literature, with high performance reported by Wei et al. (AUC 0.93) [61] and more modest results observed by Saxena et al. (AUC 0.72) [92], reflecting a high sensitivity to dataset characteristics. Investigations by Chougule et al. [65], Peng et al. [82], and Verduin et al. [85] suggest that while these signatures are detectable, they are influenced by imaging heterogeneity. However, Yu et al. [101] achieved a strong AUC of 0.923 by utilizing a more refined pipeline, indicating that improved harmonization can enhance performance.

### 4.2. Integration of Deep Learning Algorithms in Radiomics

Deep learning (DL) has shifted the radiomic paradigm from a framework centered on handcrafted feature extraction to an ecosystem driven by automated, data-driven representation learning. Initial contributions by Chang et al. [42] and Yogananda et al. [76] demonstrated that convolutional neural networks (CNNs) can directly process multi-sequence MRI to detect molecular signatures with minimal manual intervention. Subsequent evidence from Choi et al. [64], Zhang et al. [96], and Matsui et al. [70] further confirms that DL approaches consistently outperform classical radiomics in predictive accuracy.

Regarding IDH status, several CNN-based models have achieved near-perfect results. Following the landmark AUC of 0.99 reported by Yogananda et al. [76], enhancements by Pasquini et al. [81] and Chen et al. [35] confirmed that end-to-end architectures capture IDH-related imaging phenotypes with high reproducibility. Liu et al. [99] and Su et al. [104] expanded this trend by applying 2D and 3D CNNs, attention mechanisms, and multi-stream networks to extract subtle biological correlates that remain invisible to handcrafted descriptors.

Beyond standalone CNN pipelines, hybrid strategies have emerged as a highly effective paradigm. Liang et al. [97], Lin et al. [98], and Ma et al. [90] demonstrated that merging CNN-derived latent features with conventional radiomic descriptors significantly boosts performance, particularly for biomarkers with heterogeneous expression patterns such as ATRX, MGMT, or Ki-67. Supporting work by Ren et al. [59], Wei et al. [61], and Wu et al. [88] highlighted that these hybrid architectures offer resilience against inter-scanner variability while capturing complementary morphological and microstructural information.

Furthermore, advanced architectures such as Vision Transformers (ViTs), graph neural networks (GNNs), and multimodal fusion networks are being integrated into radiogenomic pipelines. Research by Meng et al. [87], Rui et al. [92], and Wang et al. [94] showed that transformer-based models leverage global contextual information, while multi-branch networks described by Zhang et al. [96] and Liang et al. [97] facilitate synergistic learning across T1, T2, FLAIR, diffusion, and perfusion sequences.

### 4.3. Radiomics Applications to Characterize the Tumor Microenvironment

The tumor microenvironment (TME)—comprising angiogenesis, hypoxia, proliferation, immune infiltration, and extracellular matrix remodeling—is pivotal in shaping glioma behavior and therapeutic response. Radiomics has emerged as a vital non-invasive tool to quantify these complex biological processes. Early contributions by Sun et al. [60], Li et al. [38], and Chaddad et al. [51] provided foundational evidence that MRI-derived features can identify vascular, proliferative, and structural signatures linked to TME dynamics. Expanding on these findings, Zhang et al. [102], Liang et al. [97], and Rui et al. [92] investigated sophisticated descriptors correlating with angiogenic pathways, cellular turnover, and immune-related microstructural heterogeneity.

Angiogenesis, a primary focus of TME research, has proven particularly amenable to quantitative imaging characterization. Investigations by Sun et al. [60], Fang et al. [78], and Zhang et al. [102] documented VEGF-associated perfusion and texture features that map onto microvascular proliferation. Similarly, perfusion-radiomics research by Calabrese et al. [86] and Wang et al. [94] suggests that subtle blood flow heterogeneity can distinguish specific vascular phenotypes across glioma subtypes. Proliferative activity has also been evaluated through Ki-67 models [37,64,97], demonstrating that radiomic analysis can effectively surrogate for cell cycle acceleration and mitotic density.

Beyond vascular and proliferative markers, several studies have linked radiomic patterns to profound microenvironmental alterations, such as chromatin remodeling and extracellular matrix disruption. Specifically, texture-based changes associated with ATRX mutation [51,59,68,88] suggest that quantitative imaging may indirectly measure the structural reorganization of tumor cell nuclei and microarchitectural disruption—features fundamentally connected to TME composition. Furthermore, research by Matsui et al. [70] and Meng et al. [87] supports the capacity of these models to reflect hypoxia-related or immune-associated spatial patterns.

### 4.4. Radiomics Integration with Multi-Omics

The integration of radiomics with multi-omics data represents a potential frontier in radiogenomics, aimed at achieving a more comprehensive biological characterization, although its clinical implementation remains in the early stages. Initial frameworks by Chaddad et al. [51] suggested that coupling radiomic features with genomic and transcriptomic signatures could enhance phenotype–genotype associations. While the volume of such studies remains selective, recent evidence from Liang et al. [97], Zhang et al. [96], and Rui et al. [92] indicates that combining MRI-derived descriptors with genomic profiles may improve the simultaneous prediction of multiple biomarkers compared to imaging-only models.

Expanding on these preliminary results, Su et al. [104], Liu et al. [99], and Lin et al. [98] explored the integration of radiomic and deep-learning representations with gene expression and mutation data, observing possible synergistic effects for biomarkers such as EGFR, IDH, and ATRX. Furthermore, contributions by Wu et al. [88], Meng et al. [87], and Ma et al. [90] have investigated the value of coupling radiomics with methylation, transcriptomic clustering, or metabolomic profiles, aiming to refine predictive models and explore biologically interpretable associations.

From a methodological standpoint, multi-omics fusion is being studied for its potential to bridge the dimensional gap between imaging and molecular data. Technical approaches using joint embedding, graph-based integration, or multi-modal variational autoencoders, as described by Wang et al. [94], are currently being evaluated to model the complex interplay between macroscopic imaging phenotypes and underlying genomic, epigenomic, or metabolic processes.

### 4.5. Discrepancy Between Clinical and Technical Quality

Our analysis revealed a discrepancy between high NOS scores and lower RQS/IBSI scores. This suggests that while the included studies are clinically robust and well-designed (high NOS), they often lack the technical transparency required in radiomics. Specifically, lower RQS reflect a lack of external validation and prospective designs, while mediocre IBSI compliance indicates insufficient reporting of image processing and feature extraction. These findings highlight that clinical rigor must be paired with stricter technical standardization to ensure the reproducibility of radiomic research.

Furthermore, the high performance reported in DL models must be interpreted with caution. Issues such as overfitting due to small sample sizes, data leakage (e.g., during image preprocessing or splitting), and class imbalance in rare molecular subtypes are frequently under-addressed. These factors can artificially inflate AUC values, masking a lack of true generalizability to real-world clinical populations.

### 4.6. Current Challenges and Future Perspectives

A significant hurdle for the clinical implementation of radiomics is reproducibility. Discrepancies in results across different centers often stem from variations in MRI acquisition protocols, divergent segmentation practices, and inconsistent feature selection. To mitigate scanner-related variability, researchers have employed techniques such as voxel and gray-level resampling, image smoothing, and test–retest analyses, while automated segmentation tools serve to minimize operator-dependent errors. Beyond technical consistency, ensuring model generalizability is vital; the inherent heterogeneity of imaging protocols and patient populations frequently restricts the applicability of models outside their original training cohorts.

Interpretability represents another substantial barrier. Although radiomics models can reach high predictive accuracy, establishing the rationale behind these outputs is a prerequisite for clinical adoption. Explainable AI (XAI) tools—including feature importance rankings, rule-based explanations, Grad-CAM, LIME, and DeepLIFT—provide essential insights into model behavior. However, correlating radiomic features with underlying biological mechanisms typically necessitates the integration of multi-omics data and close collaboration between computational scientists, radiologists, and clinicians.

Furthermore, the clinical translation of radiomics remains in its early stages. There is a notable lack of prospective trials directly evaluating its utility for glioma characterization or longitudinal treatment monitoring. While the implementation of standards like the Radiomics Quality Score (RQS) and the Image Biomarker Standardization Initiative (IBSI) can enhance methodological consistency, widespread clinical integration will require large-scale multicenter trials, standardized reporting guidelines, and interdisciplinary efforts to ensure that findings are robust, interpretable, and clinically actionable.

### 4.7. Future Integration of Radiomics and Biopsy

Although radiomics is a powerful non-invasive tool, it is unlikely to replace tissue biopsy entirely. The future of diagnosis lies in a complementary approach: biopsy remains essential for molecular analysis, while radiomics provides a full view of the entire tumor, overcoming the limits of single-tissue sampling. Together, these methods offer a more complete and accurate diagnostic profile.

### 4.8. Limitations of the Study

Significant methodological heterogeneity across the reviewed literature poses a major challenge. Variations in MRI acquisition protocols, scanner specifications, segmentation strategies, and machine learning architectures hinder direct comparisons and meta-analytic synthesis, potentially introducing bias into reported performance metrics. Furthermore, the incomplete reporting of data—specifically regarding biomarkers like PTEN and certain ATRX cohorts—limits the ability to perform robust quantitative analyses or comparative evaluations. Publication bias also remains a concern, as studies with negative or modest results are frequently underrepresented, potentially skewing the perceived efficacy of radiomic applications toward overly optimistic outcomes. Most current investigations are also limited by their retrospective, single-center design, which may restrict the generalizability of predictive models to broader clinical environments or more diverse patient populations.

Inconsistencies in image preprocessing, such as varying approaches to normalization, resampling, and smoothing, further impact feature stability and reproducibility. While deep learning (DL) models have achieved remarkable predictive accuracy, their inherent “black box” nature, coupled with the sparse use of explainable AI, obscures the biological significance of the identified features. Finally, the scarcity of external or multicenter validation remains a critical weakness, reducing overall confidence in the reliability and applicability of these models across independent datasets.

## 5. Conclusions

Radiomics is emerging as a promising non-invasive approach to characterize the molecular landscape of gliomas, offering the potential to complement traditional histopathology and guide personalized management. Among the biomarkers studied, IDH mutation prediction has consistently demonstrated the highest accuracy and reproducibility across numerous studies. Other markers, including 1p/19q codeletion, ATRX, MGMT methylation, Ki-67, and EGFR have also shown encouraging predictive performance, although results are generally more variable due to methodological heterogeneity and limited external validation. The integration of DL algorithms and multi-omics data appears particularly promising in enhancing predictive accuracy, capturing subtle imaging patterns, and linking radiomics features to underlying tumor biology.

## Figures and Tables

**Table 1:** Summary of radiomics analysis reported in each study included in the systematic review. Glioma radiogenomics has evolved from small, manual ML studies (N < 50) [35,36] to large-scale, automated Deep Learning frameworks (N > 1000). Performance has consistently improved, with AUCs for IDH, ATRX, and MGMT reaching 0.85–0.97. Software versions correspond to the most up-to-date releases at the time of publication.

Author, Year	Cohort Total (Training, Validation, Testing) (N)	MRI Sequences	Segmentation Method	Software	ML/DL Models	Molecular Pattern	Performance
Chen et al., 2017 [35]	47	DWI	NA	NA	MIMC	MGMT, IDH	Accuracy 88.47%, 77.21%
Hsieh et al., 2017 [36]	39	T1	Manual	OsiriX	Logistic regression	IDH	Accuracy 51%, 59%, 85%
Li et al., 2017 [37]	117 (78, 39, 0)	T2	Manual	MATLAB	ML	Ki-67	AUC = 0.781, accuracy 83.3% and 88.6%
Li et al., 2017 [38]	151	T1, T2, FLAIR	Automatic	CNN	ROI-only CNN	IDH	AUC = 0.80–0.96
Wu et al., 2018 [39]	102 (67, 35)	T1, FLAIR	Manual	NA	Sparse representation	IDH	Accuracy 98.5%, 94.5%
Zhang et al., 2017 [40]	152	T1, T2, FLAIR	ROI	Histogram	NA	IDH	Accuracy 82%
Chang et al., 2018 [41]	496	T1, T2, FLAIR	Manual	Matrix User, 3D Slicer	34-layer residual CNN with decision fusion	IDH	AUC = 0.90, 0.93, 0.94
Chang et al., 2018 [42]	259	T1, T2, FLAIR	Automatic	FLIRT	Automatic segmentation with 2D CNN	IDH, 1p/19q	AUC = 0.91, AUC = 0.88
Chen et al., 2018 [43]	47	T1	NA	Custom pipeline	MNMC	MGMT, IDH	AUC = 0.787, 0.886
Li et al., 2018 [44]	225	T1, T2, FLAIR	Manual, semi-automatic	Boruta	Random forest classifier	IDH	AUC = 0.96
Li et al., 2018 [45]	270 (200, 70, 0)	T2	Manual	MRIcron + pipeline MATLAB	Logistic regression model	EGFR	Training AUC = 0.90, validation AUC = 0.95
Li et al., 2018 [46]	272 (180, 92, 0)	T2	Manual	MATLAB	LASSO + SVM	p53	Training AUC = 0.896, validation AUC = 0.763
Li et al., 2018 [47]	63, 32	T2	Manual	MRIcro	SVM, LASSO	ATRX	AUC = 0.94, 0.925
Liang et al., 2018 [48]	167	T1, T2, FLAIR	Manual	M3D-DenseNet	Multi-channel ROI-only 3D DenseNet	IDH	AUC = 0.86
Lohmann et al., 2018 [49]	84	PET	VOI	NA	Logistic regression	IDH	AUC = 0.79
Lu et al., 2018 [50]	214	T1, T2, FLAIR	Manual	NA	NA	IDH, 1p/19q	AUC = 0.922–0.975
Chaddad et al., 2019 [51]	107	T1, T1-CE, T2, FLAIR	Manual	3D Slicer	Random forest	ATRX	NA
Fukuma et al., 2019 [52]	164	T1, T2, FLAIR	Manual	VOI, MATLAB	Pretrained CNN (AlexNet)	IDH	69.6% prediction accuracy
Han et al., 2019 [53]	42	T1, T2	Manual	OmniKinetics	GLCM	IDH	AUC = 0.844, 0.848
Kim et al., 2019 [54]	143	T1, T2, FLAIR	Manual	CNN	Textural, topological, and pre-trained CNN features	1p/19q	AUC = 0.71
Lewis et al., 2019 [55]	97	T1, T2	Tumor segmentation	TexRAD	Logistic regression	IDH, 1p/19q	AUC = 0.98, 0.811
Li et al., 2019 [56]	127	18-FDG PET	Manual	Elastic net	SVM	IDH	AUC = 0.911, 0.900
Li et al., 2019 [57]	NA	T1, T1-CE, T2, FLAIR	Manual	NA	ML	PTEN	NA
Nalawade et al., 2019 [58]	260	T2	NA	2D DenseNet-161 CNN	ResNET-50, DenseNET-161, inception-v4	IDH	AUC = 0.95, AUC = 0.86
Ren et al., 2019 [59]	36	NA	Manual	NA	Machine learning	ATRX	AUC = 0.93
Sun et al., 2019 [60]	239 (160, 79, 0)	T1, T2, FLAIR	Manual	NA	mRMR + SVM	VEGF	AUC Training 0.741, Validation 0.702
Wei et al., 2019 [61]	105	T1-CE, T2, FLAIR	Manual	MATLAB	ML	MGMT	Accuracy 86%, AUC 0.93
Alis et al., 2020 [62]	142 (96, 46, 0)	T1, T2 FLAIR, DWI	Manual	NA	Random forest classifier	IDH	Accuracy 86.94%
Calabrese et al., 2020 [63]	190	T1, T1-CE, T2, FLAIR	Automated	dCNN	Random forest	ATRX	AUC = 0.97
Choi et al., 2020 [64]	136	T2	Manual, automatic	ROI	Machine learning classifier	IDH	AUC = 0.90, 0.86
Chougule et al., 2020 [65]	147	T1, T2, FLAIR	Auto-encoder based automatic, manual	PyRadiomics 2.2.0	2D-CNN	IDH	NA
Decuyper et al., 2021 [66]	628, 110	T1, t1-CE, T2, FLAIR	3D U-Net automatic segmentation	NA	3D U-Net segmentation and 3D ROI extraction	IDH, 1p/19q	AUC = 0.86, AUC = 0.87
Ge et al., 2020 [67]	NA	T1, T1-CE, T2, FLAIR	CNN segmentation + 3D-2D consistency constraint	NA	Semi-supervised learning with 3D-2D consistent graph-based method and estimating labels of unlabelled data	IDH	86.53% accuracy
Haubold et al., 2020 [68]	42	T1, T1-CE, T2, FLAIR	Semi-automated	3D Slicer	SVM	ATRX	AUC = 85.1%
Lo et al., 2020 [69]	97 (69, 28)	T1	Manual	In-house software	Random forest classifier	IDH	AUC = 0.872
Matsui et al., 2020 [70]	217	T1, T2, FLAIR	NA	CNN	Deep learning model using multimodal data	IDH	58.7% accuracy
Niu et al., 2020 [71]	182	T1	Manual	A.K. software	LASSO	IDH	AUC = 0.86
Rathore et al., 2020 [72]	473	T1, T2, FLAIR	Manual, semi-automated	NA	SVM	IDH, 1p/19q, EGFR	NA
Sakai et al., 2020 [73]	100	T1, FLAIR	VOI	In-house postprocessing	XGBoost, SMOTE	IDH	AUC = 0.97, 0.95
Su et al., 2020 [74]	414	T1, FLAIR	Manual	LASSO	Logistic regression	IDH	AUC = 0.891
Sudre et al., 2020 [75]	333	T1, T2, FLAIR	Manual	Haralick texture	Random forest	IDH	Accuracy 71%
Yogananda et al., 2020 [76]	368	T2	Automatic	3D-Dense-UNet	Fully automated CNN	1p/19q	AUC = 0.953
Fan et al., 2021 [77]	157	T1, T1-CE, T2	Manual	MATLAB	Elastic Net + SVM	1p/19q	AUC 0.8079, Accuracy 0–758
Fang et al., 2020 [78]	164	T1, T1-CE, T2	Manual	pipeline MATLAB	Elastic Net + SVM	TERT	AUC 0.8446, Accuracy 0.80
Huang et al., 2021 [79]	59	T1, T2, FLAIR	Manual	NA	Logistic regression	IDH, MGMT	NA
Kihira et al., 2021 [80]	111 (91, 20, 0)	T1, T1-CE, FLAIR	Manual	LASSO	Logistic regression	IDH, ATRX, MGMT, EGFR	AUC = 1.00, 0.99, 0.79, 0.77
Pasquini et al., 2021 [81]	100	T1, T2, FLAIR	Bounding-box ROI	4-block 2D CNN	4-block 2D CNN	IDH	AUC = 0.83
Peng et al., 2021 [82]	105	T1, T2	Manual	VOI, LASSO	SVM	IDH	AUC = 0.770, 0.819, AUC = 0.747
Santinha et al., 2021 [83]	77	T1, T2, FLAIR	NA	NA	LASSO	IDH	NA
Sohn et al., 2021 [84]	418	T1, T1-CE, T2, FLAIR	Automated	A U-Net-based algorithm	Radiomics + Binary relevance	ATRX	AUC = 0.804, 0.842, 0.967
Verduin et al., 2021 [85]	185 (142, 46)	T1, T2	VOI	VASARI	XGBoost	IDH, EGFR, MGMT	AUC = 0.695, 0.707, 0.667
Calabrese et al., 2022 [86]	396	T1, T1-CE, T2, FLAIR	Semi-automated	BraTS, ITK-SNAP	CNN, Random forest	ATRX	AUC = 0.97
Meng et al., 2022 [87]	123	T1, T1-CE, T2, FLAIR	Manual	Radcloud	SVM, LASSO	ATRX	AUC = 0.93, 0.84
Wu et al., 2022 [88]	76	T1, T1-CE, FLAIR	Manual	MATLAB	Logistic regression	ATRX	C-index 0.863, 0.840
Zhong et al., 2023 [89]	329	T1, T1-CE, T2	Automated	BraTS toolkit	3D ResNet50 + C3D	ATRX	AUC = 0.953
Ma et al., 2023 [90]	459	T2	Manual, automated	ITK-SNAP, Swin transformer model	XGBoost, Random forest	ATRX	AUC = 0.8431, 0.7622, 0.7954
Medeiros et al., 2023 [91]	261	T2	Manual ROI	NA	ML	1p/19q	NA
Rui et al., 2023 [92]	23	NA	Manual	ITK-SNAP	CNN	ATRX	AUC = 0.78
Saxena et al., 2023 [93]	400 + 185	T1, T1-CE, T2, FLAIR	Subregions: ED/TC/ET	NA	Fused DL + ML (ResNet/EfficientNet + radiomiocs)	MGMT	AUC 0.75
Wang et al., 2023 [94]	82	T1, T1-CE, T2, FLAIR	Automated	BraTS	Random forest	ATRX	NA
Yang et al., 2023 [95]	133 + 27	T1, T1-CE, T2	ROI + connectomics	NA	SVM + Relief/LASSO	H3K27M	AUC 0.91
Zhang et al., 2023 [96]	102	T1, T2	Semi-automated	3D Slicer	Random forest	ATRX	AUC = 0.987, 0.975
Liang et al., 2024 [97]	309	DWI	Manual ROI	3D-Slicer	SVM	IDH, Ki-67	AUC 0.97
Lin et al., 2024 [98]	85 (61, 24)	DWI	Manual	3D Slicer	Radiomics + logistic regression monogram	ATRX	AUC = 0.97, 0.91
Liu et al., 2024 [99]	234	T1-CE, FLAIR	Manual	3D Slicer	PyRadiomics, ResNet34, Logistic regression	ATRX	AUC = 0.969, 0.956, 0.949
Yang et al., 2024 [100]	NA	T1	ROI + DWI features	NA	ML	H3K27M	NA
Yu et al., 2024 [101]	356	T1, T1-CE	NA	NA	Deep learning (CNN/Transformer)	MGMT	AUC 0.923
Zhang et al., 2024 [102]	NA	T1, T1-CE, T2, FLAIR	Whole-brain morphometry	NA	Radiomics + morphology	IDH, VEGF	NA
Niu et al., 2025 [103]	1185	T1-CE, T2 FLAIR	VOI	Deep learning	2D DL	IDH, TERT	AUC = 0.855–0.904
Su et al., 2025 [104]	204	T1-CE, T2	K-means habitat clustering	NA	SVM	IDH, EGFR	AUC = 0.943, 0.912

**Table 2:** Synthetic overview of the current literature landscape on radiomics and molecular profiling in gliomas. The table contrasts the high volume of primary data with the limited number of systematic evaluations, detailing heterogeneity in imaging protocols, segmentation methods, and predictive performance (AUC).

Feature	Systematic/Focused Evaluations	Current State of Literature (*n* = 70)
**Availability**	Limited to a few comprehensive works	Abundant individual primary studies (2017–2025)
**Biomarker Focus**	Scarce for emerging markers (H3K27M, TERT, PTEN)	High concentration on IDH (66.2%) and ATRX (36.5%)
**Methodology**	Lack of standardized cross-study protocols	High Heterogeneity: Manual segmentation (70.3%), varied MRI sequences
**Data Usage**	Limited pooled effect size or meta-analysis	Large combined cohort (*n* = 10,324), mostly retrospective
**Modeling**	Few comparative benchmarks	Dominance of SVM (39.2%) and CNNs (27.0%)
**Performance**	Variable generalizability; limited external validation	High mean AUCs (Training: 0.892; Testing: 0.842)

**Table 3:** Comparative summary of handcrafted radiomics versus deep learning-based feature extraction in the included studies.

Feature Category	Deep Learning-Based	Handcrafted Radiomics
**Feature Extraction**	Learned autonomously (Latent representations)	Predefined (Shape, First-order, Haralick/GLCM)
**Interpretability**	Lower (“Black box” nature of deep features)	Higher (Spatially and mathematically defined)
**Common Models**	CNNs (27.0%), Transformers (5.4%)	SVM (39.2%), Logistic Regression (14.9%)
**Segmentation**	Increasingly Automated/U-Net (17.6%)	Predominantly Manual (70.3%)
**Performance (IDH)**	AUC 0.88–0.99 (e.g., 3D Dense-UNet)	AUC 0.80–0.92
**Integration**	End-to-end learning (Radiomics-DL fusion)	Requires explicit feature selection (e.g., LASSO)

**Table 4:** RQS values and IBSI compliance results for each study reported in the systematic review. The transition from handcrafted radiomics to combined deep learning models correlates with improved RQS metrics and IBSI consensus adherence, addressing the primary barriers to clinical translatability in neuro-oncological imaging.

Author, Year	Method	RQS (36)	IBSI (%)
Chen et al., 2017 [35]	Handcrafted	13	43
Hsieh et al., 2017 [36]	Handcrafted	15	71
Li et al., 2017 [37]	Deep Learning	14	29
Li et al., 2017 [38]	Handcrafted	16	86
Wu et al., 2018 [39]	Handcrafted	14	57
Zhang et al., 2017 [40]	Handcrafted	15	71
Chang et al., 2018 [41]	Deep Learning	16	29
Chang et al., 2018 [42]	Handcrafted	17	86
Chen et al., 2018 [43]	Multimodal	18	57
Li et al., 2018 [44]	Handcrafted	14	71
Li et al., 2018 [45]	Deep Learning	15	29
Li et al., 2018 [46]	Handcrafted	16	57
Li et al., 2018 [47]	Combined	19	86
Liang et al., 2018 [48]	Handcrafted	15	71
Lohmann et al., 2018 [49]	Deep Learning	16	43
Lu et al., 2018 [50]	Handcrafted	14	57
Chaddad et al., 2019 [51]	Multimodal	20	86
Fukuma et al., 2019 [52]	Handcrafted	15	71
Han et al., 2019 [53]	Deep Learning	17	29
Kim et al., 2019 [54]	Deep Learning	15	43
Lewis et al., 2019 [55]	Handcrafted	14	57
Li et al., 2019 [56]	Handcrafted	16	71
Li et al., 2019 [57]	Deep Learning	17	29
Nalawade et al., 2019 [58]	Handcrafted	15	86
Ren et al., 2019 [59]	Deep Learning	16	43
Sun et al., 2019 [60]	Handcrafted	14	71
Wei et al., 2019 [61]	Handcrafted	15	86
Alis et al., 2020 [62]	Deep Learning	17	43
Calabrese et al., 2020 [63]	Multimodal	21	100
Choi et al., 2020 [64]	Handcrafted	16	71
Chougule et al., 2020 [65]	Deep Learning	15	29
Decuyper et al., 2021 [66]	Combined	18	86
Ge et al., 2020 [67]	Deep Learning	17	43
Haubold et al., 2020 [68]	Handcrafted	16	86
Lo et al., 2020 [69]	Handcrafted	15	71
Matsui et al., 2020 [70]	Deep Learning	16	29
Niu et al., 2020 [71]	Handcrafted	17	57
Rathore et al., 2020 [72]	Combined	20	86
Sakai et al., 2020 [73]	Handcrafted	15	71
Su et al., 2020 [74]	Deep Learning	16	43
Sudre et al., 2020 [75]	Multimodal	19	86
Yogananda et al., 2020 [76]	Deep Learning	17	29
Fan et al., 2021 [77]	Handcrafted	16	86
Fang et al., 2020 [78]	Deep Learning	17	43
Huang et al., 2021 [79]	Handcrafted	15	71
Kihira et al., 2021 [80]	Multimodal	19	86
Pasquini et al., 2021 [81]	Handcrafted	16	71
Peng et al., 2021 [82]	Deep Learning	17	29
Santinha et al., 2021 [83]	Handcrafted	15	86
Sohn et al., 2021 [84]	Deep Learning	16	43
Verduin et al., 2021 [85]	Multimodal	19	86
Calabrese et al., 2022 [86]	Combined	22	100
Meng et al., 2022 [87]	Deep Learning	18	43
Wu et al., 2022 [88]	Handcrafted	17	86
Zhong et al., 2023 [89]	Deep Learning	18	29
Ma et al., 2023 [90]	Handcrafted	17	71
Medeiros et al., 2023 [91]	Deep Learning	18	43
Rui et al., 2023 [92]	Handcrafted	19	57
Saxena et al., 2023 [93]	Multimodal	21	86
Wang et al., 2023 [94]	Deep Learning	18	29
Yang et al., 2023 [95]	Handcrafted	17	71
Zhang et al., 2023 [96]	Combined	22	100
Liang et al., 2024 [97]	Deep Learning	19	43
Lin et al., 2024 [98]	Handcrafted	18	71
Liu et al., 2024 [99]	Multimodal	23	86
Yang et al., 2024 [100]	Deep Learning	19	43
Yu et al., 2024 [101]	Handcrafted	18	71
Zhang et al., 2024 [102]	Combined	24	100
Niu et al., 2025 [103]	Multimodal	23	86
Su et al., 2025 [104]	Combined	25	100

**Table 5:** NOS value of each study reported in the systematic review.

Author, Year	Selection (4)	Comparability (2)	Outcome (3)	Total Score	Quality
Chen et al., 2017 [35]	3	1	3	7	High
Hsieh et al., 2017 [36]	3	2	3	8	High
Li et al., 2017 [37]	4	1	2	7	High
Li et al., 2017 [38]	4	2	3	9	High
Wu et al., 2018 [39]	3	2	3	8	High
Zhang et al., 2017 [40]	4	1	3	8	High
Chang et al., 2018 [41]	3	1	3	7	High
Chang et al., 2018 [42]	4	1	2	7	High
Chen et al., 2018 [43]	4	2	3	9	High
Li et al., 2018 [44]	3	1	3	7	High
Li et al., 2018 [45]	4	1	2	7	High
Li et al., 2018 [46]	3	1	3	7	High
Li et al., 2018 [47]	4	1	3	8	High
Liang et al., 2018 [48]	3	2	3	8	High
Lohmann et al., 2018 [49]	4	1	2	7	High
Lu et al., 2018 [50]	4	2	3	9	High
Chaddad et al., 2019 [51]	3	2	2	7	High
Fukuma et al., 2019 [52]	4	1	2	7	High
Han et al., 2019 [53]	4	1	3	8	High
Kim et al., 2019 [54]	3	2	2	7	High
Lewis et al., 2019 [55]	3	2	3	8	High
Li et al., 2019 [56]	3	2	3	8	High
Li et al., 2019 [57]	4	1	3	8	High
Nalawade et al., 2019 [58]	4	1	2	7	High
Ren et al., 2019 [59]	4	2	2	8	High
Sun et al., 2019 [60]	4	2	3	9	High
Wei et al., 2019 [61]	3	2	2	7	High
Alis et al., 2020 [62]	3	1	3	7	High
Calabrese et al., 2020 [63]	4	2	3	9	High
Choi et al., 2020 [64]	3	2	2	7	High
Chougule et al., 2020 [65]	3	2	3	8	High
Decuyper et al., 2021 [66]	4	1	2	7	High
Ge et al., 2020 [67]	4	2	3	9	High
Haubold et al., 2020 [68]	4	2	2	8	High
Lo et al., 2020 [69]	4	1	2	7	High
Matsui et al., 2020 [70]	4	2	3	9	High
Niu et al., 2020 [71]	3	2	2	7	High
Rathore et al., 2020 [72]	3	1	3	7	High
Sakai et al., 2020 [73]	3	2	3	8	High
Su et al., 2020 [74]	3	2	2	7	High
Sudre et al., 2020 [75]	3	2	3	8	High
Yogananda et al., 2020 [76]	4	2	3	9	High
Fan et al., 2021 [77]	3	2	2	7	High
Fang et al., 2020 [78]	4	1	3	8	High
Huang et al., 2021 [79]	4	1	3	8	High
Kihira et al., 2021 [80]	4	2	3	9	High
Pasquini et al., 2021 [81]	3	2	2	7	High
Peng et al., 2021 [82]	4	1	3	8	High
Santinha et al., 2021 [83]	4	1	3	8	High
Sohn et al., 2021 [84]	4	2	3	9	High
Verduin et al., 2021 [85]	3	2	2	7	High
Calabrese et al., 2022 [86]	4	1	2	7	High
Meng et al., 2022 [87]	4	1	3	8	High
Wu et al., 2022 [88]	4	2	2	8	High
Zhong et al., 2023 [89]	4	1	2	7	High
Ma et al., 2023 [90]	4	2	3	9	High
Medeiros et al., 2023 [91]	3	2	3	8	High
Rui et al., 2023 [92]	4	2	2	8	High
Saxena et al., 2023 [93]	4	2	2	8	High
Wang et al., 2023 [94]	4	2	3	9	High
Yang et al., 2023 [95]	3	2	3	8	High
Zhang et al., 2023 [96]	3	2	3	8	High
Liang et al., 2024 [97]	4	2	3	9	High
Lin et al., 2024 [98]	3	2	3	8	High
Liu et al., 2024 [99]	4	2	3	9	High
Yang et al., 2024 [100]	4	1	3	8	High
Yu et al., 2024 [101]	4	2	3	9	High
Zhang et al., 2024 [102]	4	1	2	7	High
Niu et al., 2025 [103]	3	2	2	7	High
Su et al., 2025 [104]	3	2	2	7	High

**Table 6:** Descriptive summary of methodological features and performance metrics across the analyzed literature. The table presents the distribution of NOS scores (7–9) alongside key performance indicators, noting mean AUC values for training (0.892) and testing (0.842). It details the prevalence of manual segmentation (70.3%) and the focus on specific biomarkers, such as IDH (66.2%) and ATRX (36.5%). Additionally, it categorizes the usage of Deep Learning models (27.0%) versus handcrafted radiomics.

Critical Domain	Evidence from Literature	Technical/Clinical Implications
**Methodological Rigor**	High NOS (7–9) and RQS	High reporting quality does not equate to clinical validity or biological relevance.
**Model Performance**	Training AUC (0.892) vs. Testing AUC (0.842)	Performance drops suggest overfitting or data leakage in retrospective cohorts.
**Algorithmic Trust**	DL/CNNs (27.0%) yield higher AUC (up to 0.99)	The “Black Box” nature limits clinical trust compared to interpretable handcrafted features.
**Standardization**	70.3% prevalence of manual segmentation	Significant heterogeneity hinders the reproducibility of results across different centers.
**Biomarker Scope**	High focus on IDH (66.2%) and ATRX (36.5%)	Neglect of emerging markers (H3K27M, TERT) delays comprehensive clinical adoption.

## Data Availability

Data available in a publicly accessible repository.
